# When Neurology Overlaps: Herpes Simplex Virus Encephalitis in a Patient with Progressive Multiple Sclerosis

**DOI:** 10.7759/cureus.95498

**Published:** 2025-10-27

**Authors:** Unaisah Hassan, Mirajul Islam

**Affiliations:** 1 General Medicine, King's College Hospital NHS Foundation Trust, London, GBR; 2 Internal Medicine, King's College Hospital NHS Foundation Trust, London, GBR

**Keywords:** acute encephalitis, adult neurology, herpes simplex, multiple sclerosis, patients with multiple sclerosis

## Abstract

Herpes simplex virus encephalitis (HSVE) is the most common cause of sporadic viral encephalitis in adults and carries significant morbidity and mortality if untreated. We report the case of a middle-aged female patient with progressive multiple sclerosis (MS) who developed acute gastrointestinal symptoms followed by rapid neuropsychiatric deterioration, expressive aphasia, and focal seizures. Initial evaluation suggested infectious gastroenteritis, hyponatraemia, and a possible MS exacerbation. Despite partial biochemical correction, her neurological status worsened. Later on, she developed focal seizures, which warranted urgent stroke and neurological review. Neuroimaging demonstrated temporal lobe and insular involvement (edema and hyperintensity in T2/Fluid Attenuated Inversion Recovery (FLAIR)), and cerebrospinal fluid (CSF) polymerase chain reaction (PCR) confirmed Herpes simplex virus (HSV)-1 infection. She was treated with intravenous acyclovir for 21 days. Although virological clearance and radiological resolution were achieved, she developed persistent neurocognitive sequelae, including aphasia, seizures, and personality changes. This case highlights the diagnostic complexity of HSVE in the context of MS, where overlapping features can obscure early recognition. Prompt initiation of acyclovir and multidisciplinary management are crucial to optimizing outcomes and minimizing long-term disability.

## Introduction

Herpes simplex virus encephalitis (HSVE) is a rare but serious central nervous system infection with an annual incidence of two to four cases per million [1]. Most cases in adults are due to Herpes simplex virus (HSV)-1 reactivation, with a predilection for the temporal and limbic lobes [2]. Clinically, HSVE presents with an acute onset of fever, altered mental status, focal neurological deficits, and seizures [3]. Mortality exceeds 70% if untreated, but intravenous acyclovir reduces this to less than 20% [4]. Despite treatment, approximately 50% of survivors experience long-term neurological sequelae [5].

The diagnosis of HSVE can be particularly challenging in patients with multiple sclerosis (MS), where acute neurological deterioration is often attributed to relapse, pseudo-relapse due to infection, or metabolic encephalopathy [6]. This case underscores the importance of maintaining a high index of suspicion for HSVE in MS patients with acute confusional states.

## Case presentation

A middle-aged woman with progressive MS, neurogenic bladder, and depression presented with acute confusion and vomiting. One day prior, she had nausea, vomiting, and fatigue, which was attributed to food poisoning. She also noted misnaming and speech difficulty. Prior to this admission, the patient had significant functional limitations, including reduced mobility and poor balance, relying on walking aids for short distances and a wheelchair for longer distances. She had no prior history of cognitive impairment and was not receiving any disease-modifying therapy for MS.

On arrival to the emergency department, she was disoriented, globally aphasic, and had a Glasgow Coma Scale (GCS) score of 13/15. Her vitals were stable. There was no focal motor or sensory deficit, clonus, or fasciculations. Laboratory investigations showed leukocytosis, neutrophilia, and hyponatremia (Table 1).

**Table 1:** Key laboratory and CSF results WBC, white blood cells; CRP, C-reactive protein; CSF, cerebrospinal fluid; HSV-1, herpes simplex virus type 1; PCR, Polymerase chain reaction.

Date	Test / Parameter	Result	Reference / Notes
28-04-2025	Blood tests		
	White blood cell count (WBC)	12.7 ×10⁹/L	Elevated
	Neutrophils	9.58 ×10⁹/L	Neutrophil predominant
	Haemoglobin	121 g/L	Within normal range
	Monocytes	1.70 ×10⁹/L	Mildly elevated
	Sodium (Na⁺)	123 mmol/L	Hyponatraemia
	Potassium (K⁺)	3.9 mmol/L	Normal
	Urea	5.5 mmol/L	Normal
	C-reactive protein (CRP)	<1 mg/L	Not elevated
	Plasma osmolality	259 mOsm/kg	Low
30-04-2025	Virology		
	HSV-1 DNA (PCR, CSF)	Positive	Confirmed infection
30-04-2025	CSF analysis		
	Protein	0.96 g/L	Elevated
	Glucose	3.2 mmol/L	Normal
	White blood cells	53 cells/µL	Lymphocytic predominance (51 lymphocytes, 2 polymorphs)
	Red blood cells	2 cells/µL	Minimal
	Cytology	Scattered lymphocytes and occasional macrophages; no malignant cells	Consistent with viral encephalitis
21-05-2025	Follow-up CSF analysis		
	Protein	0.78 g/L	Improved
	Glucose	3.1 mmol/L	Stable
	White blood cells	48 cells/µL	Lymphocytic predominance (48 lymphocytes, 0 polymorphs)
	Red blood cells	85 cells/µL	Slight increase, possibly traumatic tap

The CT brain was unremarkable. The working diagnosis was infectious gastroenteritis with hypovolemic hyponatremia and possible MS flare. She was managed with IV fluids, antibiotics, and electrolyte replacement.

Despite partial biochemical improvement over 24 hours, her mental state worsened with progressive confusion, restlessness, and aphasia. She later developed fever and focal seizures involving the right face and upper limb. Stroke was excluded on repeat CT and CT angiography. She was commenced on empiric IV ceftriaxone, acyclovir, and antiepileptics.

The neurology team performed a face-to-face review, noting that she was alert but significantly confused, not following commands, and exhibited repetitive speech (“yes,” “no,” “wait”). Antigravity strength was preserved in all four limbs, and there was no nystagmus, neck stiffness, or cranial nerve palsy. Reflexes and tone were grossly normal where assessable. Hoffman's and jaw jerk reflexes were absent. The neurology team recommended further investigations, including a lumbar puncture and an MRI to evaluate for possible encephalitis.

**Figure 1:**
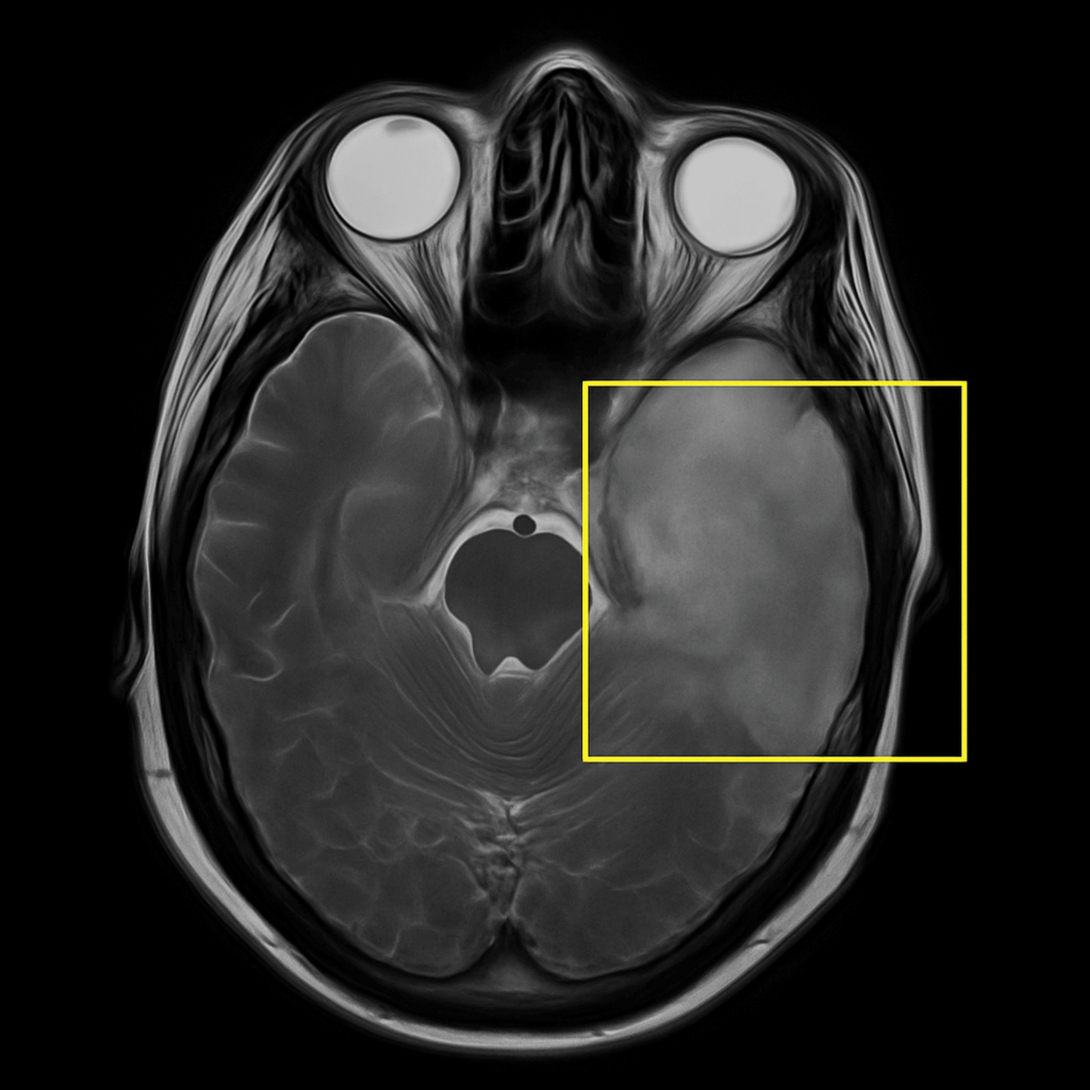
Axial T2-weighted MRI showing hyperintense signal in the left temporal lobe with loss of gray–white differentiation, consistent with herpes simplex encephalitis

The lumbar puncture demonstrated raised protein and lymphocytic pleocytosis as shown in Table 1. A contrast-enhanced CT showed cortical and subcortical low attenuation in the left temporal lobe, insula, and orbitofrontal cortex, changes consistent with HSVE. Cerebrospinal fluid (CSF) polymerase chain reaction (PCR) confirmed HSV-1 DNA. Subsequent MRI revealed a swelling in the left medial temporal lobe, hippocampus, and lingual gyrus, extending to the insula, inferior frontal lobe, and bilateral cingulate gyri, without abscess or hydrocephalus which has been shown in video 1 and figure 1 respectively.

**Video 1:** Pre-treatment MRI (axial T2-weighted video sequence) Hyperintense signal and swelling are seen in the left temporal lobe and insula, consistent with herpes simplex virus encephalitis.

**Figure 2:**
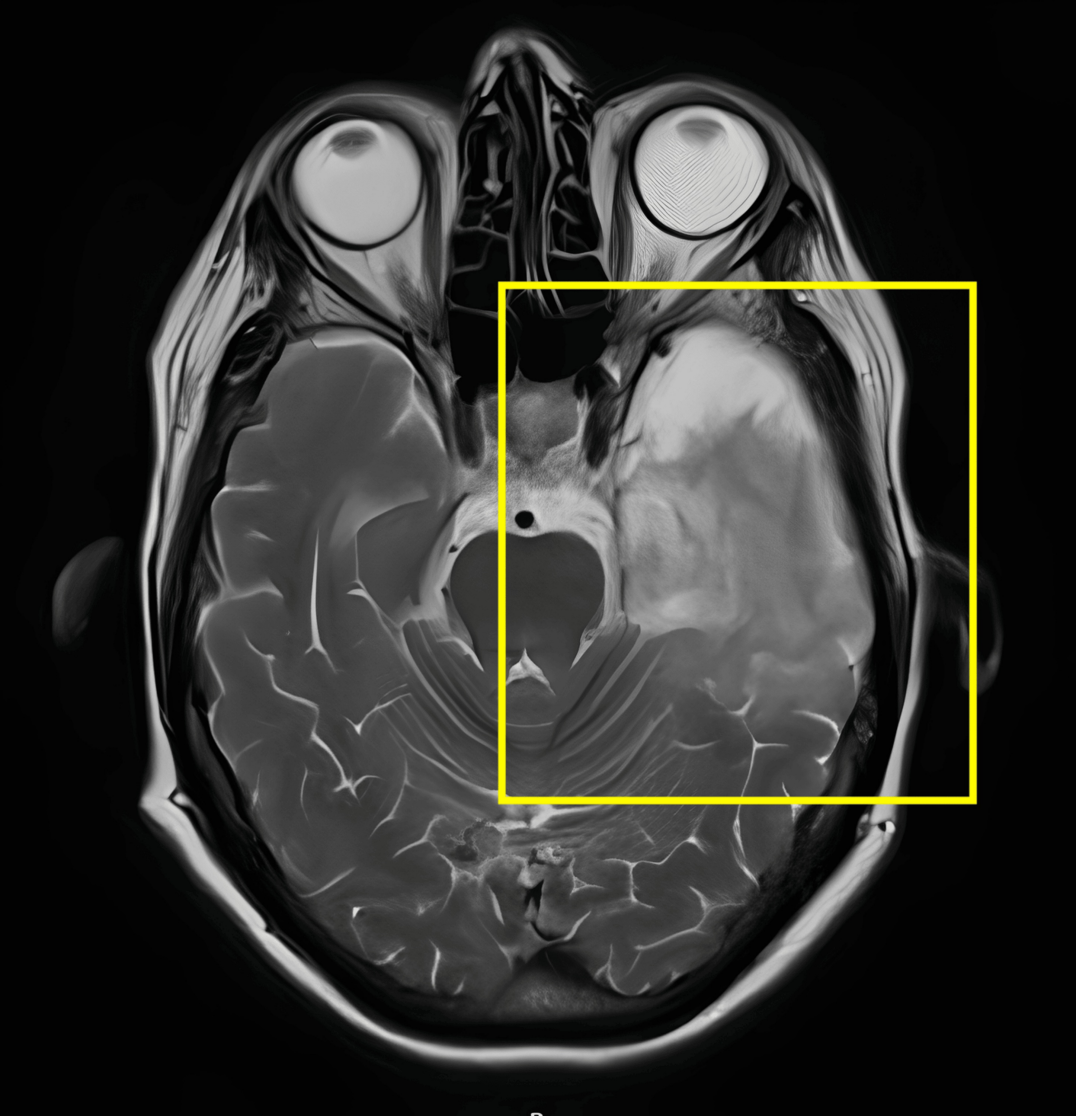
Post-treatment axial T2-weighted MRI Yellow box highlights residual hyperintensity in the right temporal lobe, showing interval improvement after treatment for herpes simplex virus encephalitis.

The patient required prolonged hospitalization complicated by recurrent seizures and urinary tract infections. She was commenced empirically on IV acyclovir prior to the availability of CSF results, which was continued for a 21-day course alongside supportive management. Follow-up MRI demonstrated partial resolution of encephalitic changes which is visualized in video 2 and figure 2 respectively.

**Video 2:** Post-treatment MRI (axial T2-weighted video sequence) Resolution of hyperintensity and swelling in the left temporal lobe and insula following completion of antiviral therapy for herpes simplex virus encephalitis.

However, she did not return to her neurological baseline after three weeks of hospital admission and was left with residual cognitive impairment, intermittent dysphagia, aphasia, seizures, and mild personality change.

## Discussion

HSVE is a neurological emergency with high morbidity, even in treated cases. Our patient presented initially with gastrointestinal illness and hyponatremia, both of which may precipitate MS pseudo-relapse. Attribution of her cognitive decline to MS progression delayed the suspicion of encephalitis [6].

Overlapping features of HSVE and MS exacerbations include cognitive and psychiatric changes mimicking fatigue or depression, aphasia and seizures resembling demyelinating lesions, and hyponatremia due to syndrome of inappropriate antidiuretic hormone secretion (SIADH), which can mislead clinicians toward metabolic encephalopathy [7].

MRI is the most sensitive imaging modality, typically revealing hyperintensity in the medial temporal and insular lobes [8], while CSF PCR remains the diagnostic gold standard with >95% sensitivity [9]. Early lumbar puncture is crucial, as confirmation guides treatment continuation [9,10].

Comparison with published literature

Previous reports have described diagnostic delays in HSVE when concurrent neurological disorders are present [6,7]. In contrast to typical cases with early fever and focal deficits, our patient initially presented with gastrointestinal symptoms and biochemical disturbances, masking the encephalitic process. Domingues et al. demonstrated that early MRI changes in HSVE localize to the temporal and insular cortices, which was consistent with our findings [8]. Similar to the study by Whitley et al., early initiation of acyclovir led to virological and radiological recovery, although neurocognitive deficits persisted [4]. This aligns with Hokkanen and Launes, who reported that nearly half of HSVE survivors exhibit long-term cognitive sequelae despite treatment [5].

Our case reinforces the need to maintain diagnostic vigilance in patients with MS and acute encephalopathy, where misattribution of symptoms to disease progression can delay antiviral therapy and worsen outcomes [6].

## Conclusions

This case highlights the diagnostic challenges of HSVE in patients with MS, where overlapping clinical and biochemical findings can delay recognition. Acute neuropsychiatric deterioration in MS is often attributed to relapse or metabolic derangements, potentially postponing essential antiviral therapy. Clinicians should maintain a high index of suspicion for HSVE in patients with MS presenting with acute confusion, aphasia, or seizures, particularly when concurrent systemic or metabolic triggers are present. Early initiation of empirical antimicrobials, alongside timely neuroimaging and CSF PCR testing, is critical for accurate diagnosis and prevention of further neurological decline.

Intravenous acyclovir remains the mainstay of treatment and significantly improves survival and neurological outcomes when administered promptly. Nevertheless, despite appropriate therapy and radiological resolution, persistent neurocognitive and behavioural sequelae are common. This case underscores the need for ongoing multidisciplinary care, including neurorehabilitation and psychological support, and reinforces the importance of vigilance and comprehensive evaluation in patients with MS with atypical or rapidly progressive neurological deterioration.
